# Supplementary material for: Microenvironmental Gradients Drive Spatial Stratification of Saccharifying Microbial Communities and Enzyme Activity in Strong-Flavor Daqu Fermentation
Source: Foods. 2025 Dec 4;14(23):4160. doi: 10.3390/foods14234160 (PMC12691976; doi:10.3390/foods14234160)
Supplement: Supplementary file 1 [file foods-14-04160-s001.zip › foods-3942752-supplementary.pdf]

Table S1

Overall statistics of strong-flavor *Daqu* metagenome.

Samples	Raw reads	Clean reads	Percent in raw reads (%)	Raw base (bp)	Clean base (bp)	Percent in raw bases (%)
0d	103614031±7613970	102602782±7345430	99±0.2	15645718731±1149709502	15374231805±1088416632	98±0.3
QP2d	91685860	90688606	98.91231429	13844564860	13503209229	97.53437082
QP 4d	88440750	87449000	98.87862778	13354553250	13052108322	97.73526735
QP 8d	104257084	103068678	98.86011966	15742819684	15373085491	97.65141061
QP 12d	110523316	108898278	98.52968762	16689020716	16186657979	96.98986091
QP 20d	98390512	97320794	98.91278338	14856967312	14518467553	97.72160932
QP 30d	89645782	88620966	98.85681626	13536513082	13157720439	97.20169706
QP 50d	98864858	97716248	98.83820194	14928593558	14623202708	97.9543227
QP 70d	106113622	104914782	98.87022987	16023156922	15546773048	97.02690377
QP 90d	108543930	107165412	98.72999071	16390133430	16064199719	98.01140294
HQ2d	119443170	118041974	98.82689316	18035918670	17546210298	97.28481603
HQ 4d	96479370	95547920	99.03456045	14568384870	14260325502	97.88542539
HQ 8d	105120172	103982048	98.91731151	15873145972	15428119427	97.19635575
HQ 12d	118392120	117308576	99.08478368	17877210120	17430130848	97.49916643
HQ 20d	91972406	91064838	99.01321707	13887833306	13599717087	97.92540555
HQ 30d	96151658	95200730	99.01101237	14518900358	14198221749	97.79130236
HQ 50d	82683866	82175456	99.38511583	12485263766	12325077420	98.7169967
HQ 70d	88739082	88214180	99.40848836	13399601382	13183633766	98.38825343
HQ 90d	87443728	86937398	99.42096476	13204002928	12981350371	98.31374956
QX2d	100559128	99860506	99.30526247	15184428328	14858920768	97.85630678

QX 4d	100293288	99607272	99.31599012	15144286488	14706168364	97.10704017
QX 8d	128214484	126746696	98.8552089	19360387084	18536782396	95.74592861
QX 12d	112869550	111868472	99.11306637	17043302050	16641927738	97.64497331
QX 20d	99419238	98769158	99.34612253	15012304938	14854147806	98.94648335
QX 30d	99152060	98539688	99.38239105	14971961060	14824383661	99.01430816
QX 50d	89159360	88608158	99.38177887	13463063360	13322182851	98.95357761
QX 70d	87113914	86536702	99.3374055	13154201014	13021153898	98.98855798
QX 90d	92208870	91693948	99.44156999	13923539370	13787556538	99.02336016
